# A Quantitative Electrophysiological Biomarker of Duplication 15q11.2-q13.1 Syndrome

**DOI:** 10.1371/journal.pone.0167179

**Published:** 2016-12-15

**Authors:** Joel Frohlich, Damla Senturk, Vidya Saravanapandian, Peyman Golshani, Lawrence T. Reiter, Raman Sankar, Ronald L. Thibert, Charlotte DiStefano, Scott Huberty, Edwin H. Cook, Shafali S. Jeste

**Affiliations:** 1 Center for Autism Research and Treatment University of California, Los Angeles Semel Institute for Neuroscience 760 Westwood Plaza, Suite A7-452 Los Angeles, California United States of America; 2 Department of Biostatistics UCLA School of Public Health Room 21-254C CHS Los Angeles, CA United States of America; 3 Department of Neurology and Psychiatry David Geffen School of Medicine 710 Westwood Plaza Los Angeles, CA United States of America; 4 Departments of Neurology and Pediatrics The University of Tennessee Health Science Center 855 Monroe Ave., Link 415 Memphis, TN United States of America; 5 Departments of Neurology and Pediatrics David Geffen School of Medicine at UCLA Los Angeles, CA United States of America; 6 Department of Neurology, Massachusetts General Hospital Harvard Medical School Boston, MA United States of America; 7 Department of Psychiatry University of Illinois at Chicago (MC 747) Chicago, IL United States of America; Universite de Lyon, FRANCE

## Abstract

**Background:**

Duplications of 15q11.2-q13.1 (Dup15q syndrome) are highly penetrant for autism spectrum disorder (ASD). A distinct electrophysiological (EEG) pattern characterized by excessive activity in the beta band has been noted in clinical reports. We asked whether EEG power in the beta band, as well as in other frequency bands, distinguished children with Dup15q syndrome from those with non-syndromic ASD and then examined the clinical correlates of this electrophysiological biomarker in Dup15q syndrome.

**Methods:**

In the first study, we recorded spontaneous EEG from children with Dup15q syndrome (*n* = 11), age-and-IQ-matched children with ASD (*n* = 10) and age-matched typically developing (TD) children (*n* = 9) and computed relative power in 6 frequency bands for 9 regions of interest (ROIs). Group comparisons were made using a repeated measures analysis of variance. In the second study, we recorded spontaneous EEG from a larger cohort of individuals with Dup15q syndrome (*n* = 27) across two sites and examined age, epilepsy, and duplication type as predictors of beta power using simple linear regressions.

**Results:**

In the first study, spontaneous beta1 (12–20 Hz) and beta2 (20–30 Hz) power were significantly higher in Dup15q syndrome compared with both comparison groups, while delta (1–4 Hz) was significantly lower than both comparison groups. Effect sizes in all three frequency bands were large (|*d*| > 1). In the second study, we found that beta2 power was significantly related to epilepsy diagnosis in Dup15q syndrome.

**Conclusions:**

Here, we have identified an electrophysiological biomarker of Dup15q syndrome that may facilitate clinical stratification, treatment monitoring, and measurement of target engagement for future clinical trials. Future work will investigate the genetic and neural underpinnings of this electrophysiological signature as well as the functional consequences of excessive beta oscillations in Dup15q syndrome.

## Introduction

Advances in genetic testing have accelerated the diagnosis of causative genetic syndromes in the context of neurodevelopmental disorders, in particular autism spectrum disorder (ASD), with the diagnostic yield of combined chromosomal microarray and whole exome sequencing of 3–20% [1–3]. In parallel, there has been an emerging interest in defining neurophysiological markers that may stratify children with ASD into biologically meaningful subgroups [4]. The convergence of discoveries in genetics and neurophysiology in ASD holds tremendous potential for the identification of biomarkers, grounded in genetic mechanisms, that can improve diagnosis, selection of treatment targets, and treatment monitoring in future clinical trials for genetically defined syndromes within the spectrum of neurodevelopmental disorders

This convergence of genetics with neurophysiology finds promise in the investigation of duplications of 15q11.2-q13.1 (or Dup15q syndrome), one of the most common copy number variants (CNVs) associated with ASD, accounting for 1–3% of cases [5]. Clinical features of Dup15q syndrome include hypotonia, global developmental delay, intellectual disability, social communication impairments, and often severe epilepsy, including infantile spasms [6–9]. Two duplication types exist: isodicentric duplications, which are characterized by two extra copies of the 15q11.2-q13.1 region of maternal origin on a supernumerary chromosome, and interstitial duplications, which can be one or more extra copies of this region on the q arm of chromosome 15 [8].

The 15q11.2-q13.1 region contains many genes critical to neural function, including *UBE3A*, a ubiquitin-protein ligase important in synaptic function [10–12], and three gamma aminobutyric acid type A receptor (GABA_A_R) subunits genes, *GABRA5*, *GABRB3*, and *GABRG3*. Recently, two studies have qualitatively noted the presence of persistent beta frequency (12–30 Hz) activity in clinical electroencephalogram (EEG) recordings from children with Dup15q syndrome [6, 7]. The clinical EEGs are quite distinctive, with spontaneous beta oscillations (SBOs) evident with visual inspection alone. These SBOs resemble the EEG effects of allosteric modulation of GABA_A_Rs by benzodiazepine drugs, even though the children for whom this observation was reported were not taking these medications [6, 7]. Since the SBOs in these studies occurred in both maternal and paternal duplication cases, it is possible that these SBOs could result from aberrant GABAergic transmission and not primarily from the maternally expressed *UBE3A* gene.

Herein, we sought to quantify EEG beta power in Dup15q syndrome to determine if it distinguished Dup15q syndrome from non-syndromic ASD and typical development. To accomplish this goal, we obtained spontaneous EEG recordings from (1) children with Dup15q syndrome, (2) age-and-IQ matched children with non-syndromic ASD and (3) age matched typically developing (TD) children. We hypothesized that spontaneous beta power would differentiate children with Dup15q syndrome from these comparison groups. In a follow-up study we examined the variation in beta power within a larger Dup15q cohort by analyzing age, duplication type, and epilepsy status as predictors of SBO strength. Given the likelihood that SBOs are related to copy number variation and seizures in Dup15q syndrome, we hypothesized that both duplication type and epilepsy would relate to spontaneous beta power.

## Subjects and Methods

### Study 1: Comparison of Dup15q syndrome with ASD and TD

#### Participants

All data were acquired in accordance with the Institutional Review Board of the University of California, Los Angeles. This study was specifically approved by the Institutional Review Board. Parents of participants provided informed written consent prior to the start of study activities. EEG datasets analyzed for this study will be deposited in a public repository following publication of this manuscript. Participants were clinically referred through the Dup15q clinic at UCLA and the national Dup15q Alliance. Children were excluded from the study if treated with medications known to pharmacologically induce beta oscillations (benzodiazepines, benzodiazepine derivatives, or barbiturates). A total of 16 participants were recruited for the first study, 5 of whom were omitted due to treatment with exclusionary medications (*n* = 2), duplication type that did not include the canonical 15q11.2-q13.1 region (*n* = 1), or insufficient length or quality of EEG recordings (*n* = 2). The remaining sample included 11 participants (5 male), 16–144 months of age (median = 54 months). Details of the sample, including age, intelligence quotient (IQ), medication, and duplication type can be viewed in Table 1. A wide age range was included to ensure that a clinically representative sample was being studied, and age matching of the comparison groups ensured that the group level comparisons would not be confounded by age differences. Both isodicentric (*n* = 8) and interstitial (*n* = 3) duplications were represented in this cohort, and 2 participants with isodicentric duplications had a diagnosis of epilepsy. Data from an ongoing study of electrophysiological biomarkers in ASD were utilized for the two comparison groups: (1) an age and IQ-matched cohort of children with non-syndromic ASD (*n* = 10) and (2) an age-matched group of TD children (*n* = 9). Preschool age children with ASD were recruited as part of a larger study investigating predictors of treatment outcome in preschoolers enrolled in a UCLA early intervention program. All children enter the program with a prior clinical diagnosis of ASD, made through the California State Regional Center, independent clinical psychologists, child psychiatrist, and/or developmental pediatricians. Diagnoses were confirmed by UCLA psychologists based on DSM-IV criteria. Non-syndromic ASD was defined by normal clinical chromosomal microarray testing, but most children had not undergone whole exome sequencing. Details of both comparison groups are available in Table 1.

**Table 1:** Dup15q syndrome participant characteristics. Older participants tested in Orlando did not undergo cognitive testing, as age-appropriate cognitive tests were not available. N/A = not available.

Group	Age (months)	Site	Gender	Meds	Genetics	Epilepsy	Verbal developmental quotient (VDQ)	Nonverbal developmental quotient (NVDQ)
Dup15q	99.9	UCLA	Female	risperidone	isodicentric	no	43	47
Dup15q	42.5	UCLA	Male	none	isodicentric	no	12	32
Dup15q	44.5	UCLA	Male	none	isodicentric	no	72	64
Dup15q	28.1	UCLA	Female	none	isodicentric	no	39	46
Dup15q	42.8	UCLA	Female	levetiracetam	isodicentric	yes	6	4
Dup15q	55.9	UCLA	Female	none	interstitial	no	105	100
Dup15q	230.1	UCLA	Female	zoloft	interstitial	no	N/A	56
Dup15q	54.2	UCLA	Male	none	interstitial	no	8	24
Dup15q	57	UCLA	Male	none	isodicentric	no	48	46
Dup15q	143.8	UCLA	Female	levetiracetam	isodicentric	yes	7	12
Dup15q	106.1	UCLA	Male	no anticonvulsant	interstitial	no	33	34
Dup15q	15.8	UCLA	Female	no anticonvulsant	isodicentric	no	48	79
ASD	61.2	UCLA	Male	Focalin, risperdone	N/A	no	51	48
ASD	26.6	UCLA	Male	none	N/A	no	19	45
ASD	39.3	UCLA	Male	none	N/A	no	33	46
ASD	63	UCLA	Male	none	N/A	no	43	41
ASD	28.8	UCLA	Male	none	N/A	no	28	52
ASD	53.2	UCLA	Female	risperidone	N/A	no	56	51
ASD	48.6	UCLA	Male	none	N/A	no	17	49
ASD	32.3	UCLA	Male	none	N/A	no	25	43
ASD	58.5	UCLA	Female	zoloft	N/A	no	103	74
ASD	98.7	UCLA	Male	melatonin	N/A	no	21	50
TD	57	UCLA	Female	none	N/A	no	122	94
TD	54	UCLA	Male	none	N/A	no	98	97
TD	29	UCLA	Male	none	N/A	no	131	157
TD	55	UCLA	Male	none	N/A	no	140	107
TD	38.8	UCLA	Female	none	N/A	no	145	149
TD	43.8	UCLA	Male	none	N/A	no	109	115
TD	40.8	UCLA	Male	none	N/A	no	141	113
TD	59.6	UCLA	Female	none	N/A	no	127	103
TD	59.6	UCLA	Male	none	N/A	no	112	117
Dup15q	93.2	Orlando	Male	rufinamide 1200 mg b.i.d., levetiracetam 2000 mg b.i.d., lacosamide 40 mg b.i.d., Epidiolex (canibidiol) 7 mL x2	isodicentric	yes	39	37
Dup15q	108.2	Orlando	Male	no anticonvulsant	interstitial	no	N/A	N/A
Dup15q	147.3	Orlando	Female	lamotrigine 300 mg b.i.d. lacosamide 100mg b.i.d. felbamate 1200mg/800mg/1000mg	isodicentric	yes	14	N/A
Dup15q	110.5	Orlando	Male	no anticonvulsant	isodicentric	no	12	21
Dup15q	117.5	Orlando	Female	no anticonvulsant	interstitial	no	37	44
Dup15q	65.45	Orlando	Male	levetiracetam 500mg AM 750mg PM lamotrigine 100 mg b.i.d.	isodicentric	yes	9	13
Dup15q	44.9	Orlando	Female	no anticonvulsant	interstitial	no	11	27
Dup15q	47	Orlando	Female	no anticonvulsant	isodicentric	no	N/A	N/A
Dup15q	81.2	Orlando	Male	valproic acid 250mg b.i.d. oxcarbazepine 300mg b.i.d.	isodicentric	yes	N/A	N/A
Dup15q	48.7	Orlando	Male	no anticonvulsant	isodicentric	no	N/A	N/A
Dup15q	384	Orlando	Female	valproic acid	interstitial	yes	N/A	N/A
Dup15q	161.6	Orlando	Male	no anticonvulsant	interstitial	no	N/A	N/A
Dup15q	29.4	Orlando	Male	no anticonvulsant	isodicentric	no	36	29
Dup15q	111.7	Orlando	Female	no anticonvulsant	interstitial	no	26	24
Dup15q	86	Orlando	Female	no anticonvulsant	isodicentric	no	N/A	N/A

#### Clinical assessment

Owing to the large range in age and developmental ability amongst participants in our study, several assessments were used to evaluate cognition, language, and motor skills. The following measures were used to match participants by cognitive function: the Mullen Scales of Early Learning (MSEL)[13], the Stanford Binet Intelligence Scales-Fifth Edition (SB5) [14], the Differential Ability Scales Second Edition (DAS-II)[15], Preschool Language Scales-Fifth Edition (PLS-5) [16], and the Leiter International Performance Scales–Revised (Leiter-R) [17].

#### EEG recording

Spontaneous EEG was recorded at 500 Hz using high-density 129 channel geodesic nets with Ag/AgCl electrodes (Electrical Geodesics, Inc., Eugene, OR, USA) while participants watched nonsocial silent videos of bouncing soap bubbles and other abstract images on a computer monitor for 2 to 6 minutes, depending on the child’s level of compliance with the paradigm. No sedation was employed for electrode placement or EEG recording. EEG signals were amplified using a Net Amps 300 amplifier (Electrical Geodesics, Inc., Eugene, OR, USA) with a low-pass analog filter cutoff frequency of 6 KHz. EEG signals were vertex-referenced at the time of recording and later re-referenced to average after preprocessing and artifact reduction.

#### Data processing

EEG recordings were band pass filtered at 1–50 Hz using a finite impulse response (FIR) filter with the EEGLAB toolbox [18]. Recordings were then segmented into 1000 ms segments for preprocessing. Noisy or loose channels were spherically interpolated using EEGLAB, and EEG recording segments with more than 11 interpolated channels were rejected. All remaining segments were manually inspected for non-stereotyped artifacts, e.g., electromyogram (EMG), and rejected based on qualitative inspection. Following manual artifact rejection, a combined principal component analysis (PCA) and independent component analysis (ICA) approach was used to eliminate stereotyped artifacts, e.g., ocular artifacts. PCA was performed prior to ICA to reduce each dataset to 24 dimensions, an important consideration for successful extraction of meaningful independent components (ICs) from EEG recordings [19]. ICs corresponding to physiological artifact were subtracted from EEG recordings. All EEG recordings were re-referenced to an average reference prior to power calculations.

EEG recordings from ASD and TD cohorts were acquired and processed according to the same protocol described above with the sole discrepancy that 8 ASD and 9 TD recordings were sampled at a lower frequency (250 Hz) than those with Dup15q syndrome. Accordingly, EEG signals from all groups were downsampled to 250 Hz prior to spectral analysis if originally sampled at 500 Hz to ensure that all power spectral densities (PSDs) are computed with the same frequency resolution.

#### Statistical analysis of spectral power

**Fig 1:**
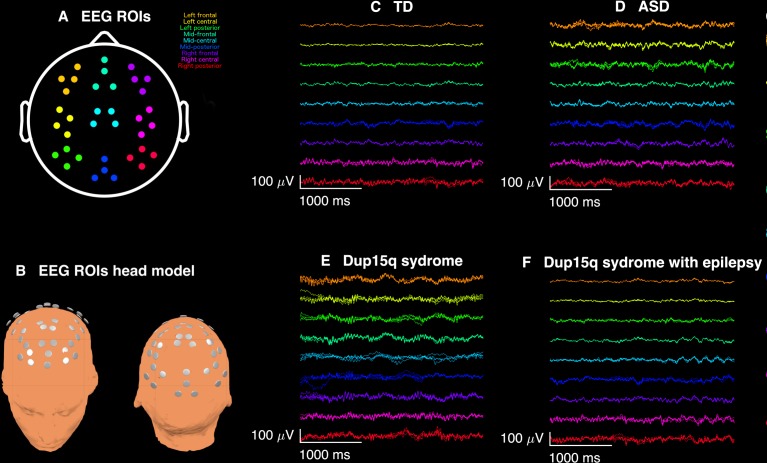
Broadband EEG recordings. Qualitative analysis of spontaneous electroencephalogram (EEG) recordings from participants with Dup15q syndrome revealed overt beta frequency oscillations apparent upon visual inspection. (A) EEG recordings from 9 scalp regions of interest (ROIs) were analyzed: left frontal (orange), left central (yellow), left posterior (green), mid-frontal (aquamarine), mid-central (cyan), mid-posterior (blue), right frontal (purple), right central (pink), right posterior (red). (B) 3-dimenisonal head model showing ROI electrode locations. (C) 3 s of broadband EEG recordings from a representative 29-month-old TD child from 36 channels across 9 ROIs. (D) Same duration of EEG recorded from a 27-month-old child with nonsyndromic ASD. (E) EEG from a representative Dup15q syndrome participant (age 28 months) reveals spontaneous beta oscillations (SBOs) in virtually all channels and all ROIs. The overt quality of SBOs likely allows for their easy detection in clinical EEG recordings. By contrast, (F) a 43-month-old participant with both Dup15q syndrome and epilepsy does not show nearly such distinct SBOs. It is possible that beta activity is reduced in children with both Dup15q syndrome and epilepsy.

To study spectral power, 9 regions of interest (ROIs) were defined corresponding to the locations of channels F3, Fz, F4, C3, Cz , C4, P3, Pz, P4 in the international 10–20 montage, as per prior studies by our group [20]. Each ROI consisted of 4 electrodes and was chosen to allow for maximum spatial coverage of the scalp (see Fig 1A). For each electrode, PSDs were computed according to Welch’s method [21] using the pwelch function in MATLAB. This method divides the signal into sections of equal length with 50% overlap and uses a Hamming window to estimate a modified periodogram for each segment. Segments are overlapped by 50% owing to the fact that the Hamming window weighs the center of the data segment more strongly than the sidelobes, which are attenuated by 42.5 dB. Periodograms for all segments are then averaged to provide a final spectral estimate. Relative power was then calculated for specific frequency bands, and these relative power values were then averaged across a particular ROI. 2000 ms of signal sampled at this rate were zero-padded to create 512-point Hamming windows to compute PSDs with approximately 0.5 Hz frequency resolution. The 2000 ms signal length was chosen as twice the period of the slowest oscillation examined (1 Hz delta activity). The following frequency bands were examined separately: delta (1–4 Hz), theta (4–8 Hz), alpha (8–12 Hz), beta1 (12–20 Hz), beta2 (20–30 Hz), and gamma (30–48 Hz). EEG power was calculated from PSDs as relative power, i.e., the proportion of total (1–48 Hz) spectral power accounted for by a given frequency band. Normalizing spectral power in this manner allows for meaningful comparisons between subjects with different overall levels of signal power.

Relative power values from 6 frequency bands and 9 ROIs were analyzed using repeated measures analysis of variance (ANOVA), with multiple testing corrected for using false discovery rates (FDR). The final model for each band included group (Dup15q, ASD, TD) and ROI main effects. Because no significant group-by-ROI interactions were found, interactions were removed from the final model. Since estimated variances for relative power in the Dup15q group were larger compared to ASD and TD groups, especially for beta power, separate group variances and within-subject correlations were allowed for in the fitted repeated measures ANOVA models. Comparisons of spectral power were performed both with and without outliers (defined by being more than three standard deviations from the mean) in order to assess the extent to which comparisons might be biased by outliers.

### Study 2: Subgroup analyses of EEG power within Dup15q syndrome

#### Participants

All data were acquired in accordance with the Institutional Review Board of the University of California, Los Angeles. This study was specifically approved by the Institutional Review Board of the University of California, Los Angeles. Parents of participants provided informed written consent prior to the start of study activities. After this initial study, we expanded the cohort in order to examine the variability in beta power within Dup15q syndrome. Spontaneous EEG data were acquired from individuals at the 2015 National Dup15q Alliance conference in Orlando, Florida, with consenting procedures as described in the first study. EEG datasets analyzed for this study will be deposited in a public repository following publication of this manuscript.

#### EEG recording and processing

EEG recording followed the protocol described in the initial cohort study. A total of 24 participants underwent EEG recordings at the Orlando site, with 9 participants omitted due to treatment with exclusionary medications or insufficient length or quality of EEG recordings. Mean beta power did not differ between testing sites using Welch two-sample *t*-tests (beta1 power: *t =* 0.41, *p* = 0.69; beta2 power: *t* = 0.68, *p* = 0.50). Therefore, the data acquired from the Orlando site were combined with the 11 participants in the initial Dup15q cohort and 1 additional adult tested at UCLA, resulting in a final sample of 27 participants, 16–384 months of age (median = 81.2 months). As with the first study, no sedation was employed for electrode placement or EEG recording. Table 1 provides details about this cohort. EEG data were processed according to the protocol described in the initial study.

#### Statistical analysis of spectral power

To model the effects of age, duplication type, and epilepsy on beta power, several simple linear regression models were implemented using two outcome measures, beta1 power and beta2 power, averaged across all ROIs. Simple linear regressions were used, with each variable as a separate predictor of beta1 and beta2 power. Age was treated as a continuous variable while duplication type and epilepsy were treated as binary variables. Prior to regression modeling, Welch two-sample *t*-tests were used to test for differences in mean beta power between testing sites. Having tested the null hypothesis that means of outcome measures do not differ between testing sites, three univariate regressions were performed for beta1 power and beta2 power using the aforementioned predictors.

## Results

### Study 1 –Comparison of Dup15q syndrome with ASD and TD

**Fig 2:**
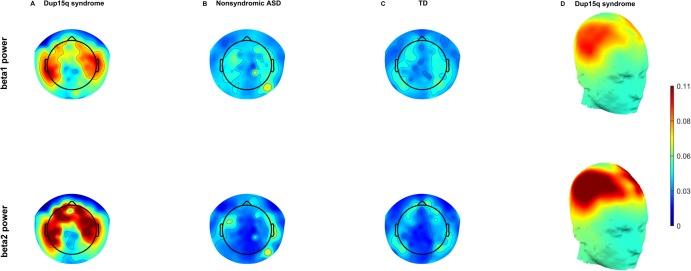
Group averaged EEG topoplots. Topographic scalp plots of relative beta1 (12–20 Hz) power (row 1) and relative beta2 (20–30 Hz) power (row 2) averaged across all participants. Three groups are compared, Dup15q syndrome group (A), nonsyndromic ASD group (B), and TD group (C). Qualitative inspection of these scalp plots reveals profound differences in relative power between Dup15q syndrome and comparison groups. This contrast is most pronounced in the beta2 band. Beta power in both subbands is most pronounced over frontotemporal scalp regions and attenuated over the midline. This is best visualized in the Dup15q syndrome cohort by projecting averaged power onto a generic 3-dimensional head model (D).

Results of behavioral testing, along with duplication type and epilepsy history, are summarized for all participants in Table 1. Qualitative analysis of bandpass filtered EEG traces from several participants with Dup15q syndrome revealed fast sinusoidal oscillations at beta frequencies apparent upon visual inspection. Fig 1 shows beta activity from 9 scalp regions of interest (ROIs) in a 29-month old TD child (Fig 1C), a 27-month-old child with nonsyndromic ASD (Fig 1D), a 28-month-old child with Dup15q syndrome (Fig 1E), and a 43-month old participant with both Dup15q syndrome and epilepsy (Fig 1F). ROIs were selected for maximum scalp coverage and their correspondence to 10–20 montage channels [20]. Visual inspection of topographic scalp plots for both beta1 (12–20 Hz) relative power and beta2 (20–30 Hz) relative power averaged across participants revealed a diffuse pattern of beta activity in Dup15q syndrome that appeared strongest over frontotemporal regions (Fig 2), in stark contrast to both comparison groups. Frontotemporal distributions of beta power in Dup15q syndrome are best visualized by projection onto 3-dimensional head models (Fig 2D).

Because no significant group-by-ROI interactions were found in modeling power in each frequency band, group means were averaged across regions, and comparisons were made between (1) the Dup15q cohort and the ASD cohort and (2) the Dup15q cohort and the TD cohort, resulting in a total of 12 frequency comparisons (2 pairwise comparisons for 6 frequency bands, see Table 2). Group comparisons were robust to the removal of outliers greater than 3 standard deviations from the mean, and thus outliers were included in the final analysis.

**Table 2:** Results from contrast analysis following repeated measures ANOVA. 12 pairwise comparisons were performed following the repeated measures analysis of variance (ANOVA), one comparison of duplication 15q11.2-q13.1 (Dup15q) syndrome to the nonsyndromic ASD group and one comparison of Dup15q syndrome to the TD group for each of 6 frequency bands. Mean estimate is the mean difference between groups. All comparisons were corrected for multiple testing using false discovery rates (FDR). Statistically significant comparisons appear in bold.

Frequency	Comparison	Mean estimate	*t* statistic	p-value	FDR adj.
Delta	Dup15q - ASD	**-0.089**	**-2.51**	**0.013**	**0.025**
	Dup15q - TD	**-0.094**	**-2.67**	**0.0081**	**0.024**
Theta	Dup15q –ASD	0.0072	0.23	0.82	0.82
	Dup15q - TD	0.024	0.79	0.43	0.47
Alpha	Dup15q –ASD	-0.024	0.011	0.036	0.055
	Dup15q - TD	-0.027	0.015	0.078	0.10
Beta1	Dup15q –ASD	**0.026**	**2.57**	**0.011**	**0.025**
	Dup15q –TD	**0.026**	**2.75**	**0.0065**	**0.024**
Beta2	Dup15q –ASD	**0.066**	**4.15**	**<0.0001**	**0.0006**
	Dup15q –TD	**0.062**	**4.02**	**<0.0001**	**0.0006**
Gamma	Dup15q - ASD	**0.015**	**2.31**	**0.022**	**0.038**
	Dup15q - TD	0.0078	1.18	0.24	0.29

**Fig 3:**
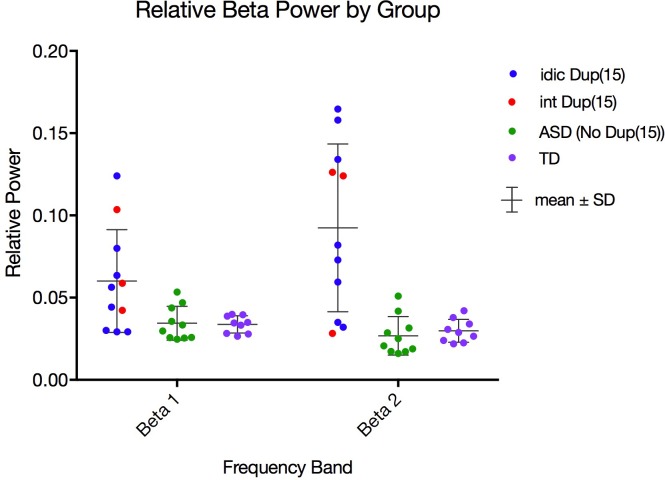
Distributions of relative beta1 and beta2 power for all groups. Dot plots of relative beta1 (12–20 Hz) power (A) and beta2 (20–30 Hz) power (B) averaged across all ROIs in the Dup15q syndrome group (left), nonsyndromic ASD group (center), and TD group (right). Participants with interstitial duplications [int Dup(15)] are colored red, while participants with isodicentric duplications [idic Dup(15)] are colored blue. Comparison group participants with nonsyndromic ASD are colored green, while children in the TD comparison group are colored purple. The Dup15q cohort features greater mean and standard deviation (mean ± S.D.) in beta1 power (0.060 ± 0.031) and beta2 power (0.092 ± 0.051) in relation to comparison groups [beta1 power: 0.034 ± 0.010 (ASD), 0.034 ± 0.0052 (TD); beta2 power: 0.027 ± 0.012 (ASD), 0.030 ± 0.0070 (TD)].

Effect sizes were computed from raw data averaged across ROIs using Cohen’s *d*. Large effect sizes were found in both beta1 power (Dup15q –ASD, *d* = 1.08; Dup15q –TD, *d* = 1.12) and beta2 power (Dup15q –ASD, *d* = 1.73; Dup15q –TD, *d* = 1.63). These are especially large effect sizes for studies of EEG power in neurodevelopmental disorder. The Dup15q cohort was best distinguished from comparison groups in the beta2 band, in which participants exhibited significantly stronger spontaneous power than both the ASD (*p* = 6 × 10^−4^, FDR corrected) and TD (*p* = 6 × 10^−4^, FDR corrected) groups (see Table 2 and Fig 3). Children with Dup15q syndrome also exhibited stronger spontaneous beta1 power (Fig 3) than both the ASD group (*p* = 0.0252, FDR corrected) and the TD group (*p* = 0.02, FDR corrected). Two other significant findings included weaker spontaneous delta power in Dup15q syndrome compared to both the ASD group (*p* = 0.03, FDR corrected; *d* = -1.07) and the TD group (*p* = 0.02, FDR corrected; *d* = -1.13) and higher spontaneous gamma power in Dup15q syndrome compared to the ASD group (*p* = 0.04, FDR corrected; *d* = 0.97). Spontaneous gamma power was not significantly different between children with Dup15q syndrome and the TD group.

These data suggest that a pattern of high beta power and low delta power distinguishes Dup15q syndrome from both children with nonsyndromic ASD and TD children, with the largest effect size found in the beta2 band. High gamma power may also distinguish Dup15q syndrome from nonsyndromic ASD. We next asked if gamma power is related to beta2 power, possibly as an extension of the same broadband electrophysiological activity, by adding EEG recordings from Study 2 to examine a larger Dup15q syndrome cohort (*n* = 27). In fact, beta2 power and gamma power were significantly correlated in every cohort (Dup15q syndrome, *r* = 0.43, *p* = 0.024; non-syndromic ASD, *r* = 0.78, *p* = 0.0080; TD, *r* = 0.88, *p* = 0.0017), suggesting that gamma power and beta2 power reflect the same electrophysiological process. Given the reciprocal pattern of high beta power and low delta power in Dup15q syndrome, we then asked a similar question about how these two frequency bands might be related. We observed that delta power and beta power are significantly related only in the Dup15q cohort (beta1, *r* = -0.63, *p* = 5 × 10^−3^; beta2, *r* = -0.47, *p* = 0.013) and are thus possibly codependent features of the same EEG signature. This relationship between delta power and beta power was not significant in either comparison group, but appeared as a trend in the ASD cohort (beta1, *r* = -0.62, *p* = 0.056; beta2, *r* = -0.62, *p* = 0.056).

**Fig 4:**
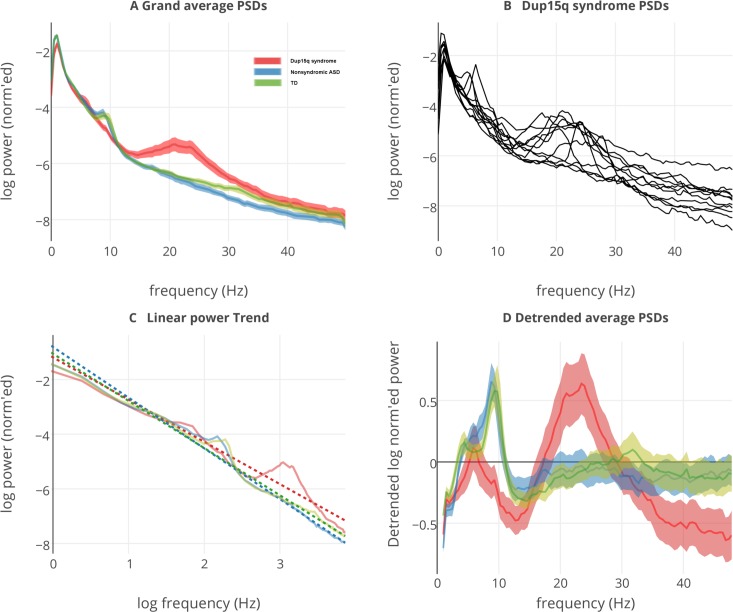
Grand averaged power spectral densities from all groups. (A) Power spectral densities (PSDs) averaged across all regions of interest (ROIs) and participants for the Dup15q syndrome group (red), nonsyndromic ASD group (blue), and TD group (green). Before averaging, participant PSDs are normalized such that the area under the curve equals 1 to emphasize relative power. Translucent highlights represent standard error of the mean (SEM) computed across participants. An enormous peak from 12–30 Hz reveals the presence of powerful spontaneous beta oscillations (SBOs) in the Dup15q cohort. PSDs are normalized to represent relative power. (B) Individual PSDs, averaged across ROIs, from participants with Dup15q syndrome. (C) Group averaged linear trends (dotted lines) fitted from log-log transformed PSDs. Linear trends represent the 1/*f* distribution inherent in the EEG. (D) Group averaged PSDs with linear trends removed to emphasize deviations from the 1/*f* trend. Dup15q syndrome shows the largest deviation, with a peak frequency (~23 Hz) in the beta band. Both comparison groups feature peak frequencies in the alpha band.

While spectral power measures are integrated across frequency bins, they do not capture peaks and valleys in the frequency domain. To capture such spectral features, grand averaged PSDs were visualized in Fig 4A by computing the mean across ROIs and all participants. A clear spectral peak in the beta band can be seen for the Dup15q syndrome cohort studied at UCLA in Fig 4A. Fig 4B shows the presence of beta spectral peaks in PSDs averaged across ROIs for individual participants with Dup15q syndrome. After removing the 1/*f* trends (dotted lines) that account for most variance in EEG PSDs (Fig 4C), the peak frequency for participants with Dup15q syndrome (~23 Hz) was higher than that of both the ASD (~8 Hz) and TD (~9 Hz) comparison groups (Fig 4D).

### Study 2 –Within-group analysis

**Fig 5:**
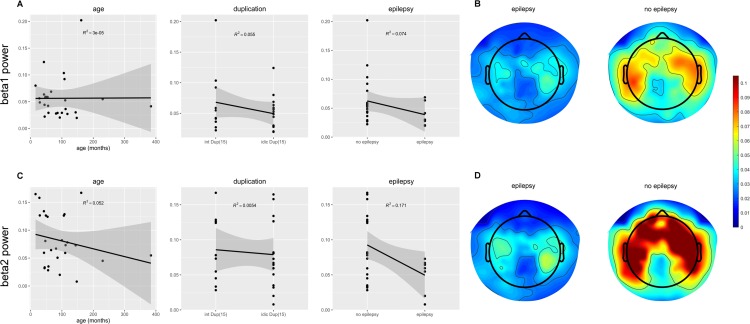
Age, duplication type, and epilepsy as predictors of beta1 and beta2 power. (A) Scatter plots of age, duplication type, and epilepsy against relative beta1 (12–20 Hz) power. Duplication type and epilepsy are treated as binary variables. Interstitial duplications and no epilepsy are represented as 0; Isodicentric duplications and epilepsy are represented as 1. Highlighted area around regression line represents the 95% confidence region. (B) Topographic scalp plots of beta1 power averaged across participants with epilepsy (left) and without epilepsy (right). (C) Scatter plots of age, duplication type, and epilepsy against relative beta2 (20–30 Hz) power. (D) Topographic scalp plots of beta2 power averaged across participants with epilepsy (left) and without epilepsy (right). The relationship between epilepsy and beta2 power is statistically significant (*R*^2^ = 0.17, *p* = 0.032).

Of the regression models tested, only epilepsy diagnosis statistically predicted beta2 power, with stronger beta2 power in participants with Dup15q syndrome who did not have epilepsy (*R*^2^ = 0.17, *p* = 0.03; Fig 5). Qualitative evidence for this finding can be seen in averaged scalp plots of beta power in individuals with Dup15q with and without epilepsy (Fig 5B and 5D). Neither age nor duplication type significantly predicted beta power within the Dup15q cohort. Because between group differences were also found in delta power and gamma power, we asked if these variables could also predict epilepsy status. Neither delta power nor gamma power significantly predicted epilepsy status in Dup15q syndrome, although a trend level finding is observed for gamma power (*R*^2^ = 0.12, *p* = 0.076).

## Discussion

Dup15q syndrome is highly penetrant for intellectual disability, epilepsy, and ASD. Several clinical reports have described a distinctive feature on clinical EEG that may represent an electrophysiological biomarker of this syndrome in the form of increased beta oscillations. We found that EEG beta power (SBOs) most strongly distinguished children with Dup15q syndrome from both (1) age/IQ-matched children with nonsyndromic ASD and (2) age-matched TD children. Changes in the EEG beta band were qualitatively obvious upon visual inspection of data as SBOs. These SBOs, as measured by power in the beta1 and beta2 bands, correlate with epilepsy diagnosis but not age or duplication type. Although our clinically referred sample included a small cohort with a relatively large age range, the robustness of this EEG signature in both this cohort and the larger sample examined in the second study is evident by the large effect sizes (|*d*| > 1).

### The promise of biomarkers

There has been a tremendous interest in the field of neurodevelopmental disorders in the identification of quantitative measures of brain function that may relate to specific genetic etiologies, as these “biomarkers” can help provide clues into the neurobiological sequelae of a genetic variation (linking genes to brain) and shed light on the impact of aberrant brain function on behavior (linking brain to behavior). A quantitative measure of neural function in a genetically defined subgroup may provide a more refined assay of subtle individual differences that can inform predictors of outcome, particularly in the context of interventions that target the specific mechanism underlying the measure. In other words, one may see a change in a biomarker with treatment that precedes any overt behavioral change but that suggests engagement of the biological target and, therefore, hope for clinical improvement.

For both practical and scientific reasons, EEG is a particularly robust method to measure neural function in developmental disorders. Not only does it have excellent motion tolerance, but its temporal resolution also allows it to resolve neurophysiological oscillations and dynamics on a millisecond scale. Power Spectral Densities (PSDs) from EEG recordings follow a characteristic 1/*f* distribution, so named because spectral power is inversely proportional to frequency. 1/*f* distributions are ubiquitous in the brain and are a likely signature of balance between neural excitation and inhibition (E/I balance) [22, 23].

### Presumed mechanisms of spontaneous oscillations in Dup15q syndrome

Enhanced SBOs and diminished delta oscillations observed in Dup15q syndrome represent deviations from the 1/*f* distribution (Fig 4C). Because E/I balance is believed to be necessary for varied and complex electrophysiological signals [22, 24], deviations from the 1/*f* distribution likely represent a disruption of balanced neurotransmission. In Dup15q syndrome, a disruption of E/I balance could be created by GABA_A_R subunit gene overexpression. SBOs observed in Dup15q syndrome [6, 7] strongly resemble those induced by positive allosteric modulators (PAMs) of GABA_A_Rs such as benzodiazepines and barbiturates [25–27]. Benzodiazepines, barbiturates, and other GABA_A_R PAMs increase the net chloride flux through the GABA_A_R’s ion pore [28, 29]. Barbiturates [28, 30] and at least one benzodiazepine compound, zolpidem [31, 32], have been shown to increase the time constant of GABA_A_Rs by lengthening the duration of hyperpolarizing chloride currents through the receptor’s ion pore. Beta activity resulting from GABA_A_R modulation or other dysfunction may actually represent slowed gamma activity, as the GABA_A_R time constant is known to control the frequency of gamma oscillations [24]. In the healthy brain, inhibitory interneurons with reciprocal connections fire synchronously to inhibit pyramidal cells, silencing themselves in the process. Pyramidal cells recover from inhibition to enjoy a period of excitability before being silenced again by interneurons, beginning the gamma cycle anew [24, 33]. Lengthening the time constant of GABA_A_Rs through altered GABA_A_R gene expression would lengthen the period of the gamma cycle to that characteristic of beta oscillations. Together, high-frequency beta and gamma oscillations are hypothesized to play a critical role in temporal binding of local circuits during cognitive tasks.

In addition to elevated beta power, spectral anomalies were also observed in the delta and gamma bands of EEG recordings from participants with Dup15q syndrome. It is possible that reduced delta power observed in Dup15q syndrome may be linked to enhanced beta power in a reciprocal manner. A significant negative correlation was observed between delta power and both beta1 power and beta2 power only in the Dup15q syndrome cohort. Angelman syndrome, most commonly caused by maternal deletion of the 15q11-q13 region, including *UBE3A*, features enhanced delta oscillations in clinical EEG recordings [34, 35], suggesting a reciprocal relationship between deletion and duplication of the GABA_A_R subunits. Furthermore, loss of *UBE3A* has been associated with enhanced delta oscillations [36] and suppression of ventral striatal GABA co-release in mouse models of Angelman syndrome [37], underscoring the relationship between the ubiquitin ligase and GABAergic transmission.

Evidence from pharmacological studies also suggests that reduced delta power in Dup15q syndrome may be directly related to GABA_A_R subunit gene overexpression rather than *UBE3A per se*. For instance, the benzodiazepine compounds diazepam and zolpidem decrease cortical EEG delta power in awake, behaving rats [27, 38]. Similarly, midazolam has been shown to reduce EEG delta power during sleep in rats [39]. In humans with generalized anxiety disorder, the benzodiazepine clorazepate has been shown to reduce delta power in scalp EEG recordings [40]. All studies considered so far also associated increased beta power with benzodiazepine challenge. The foregoing evidence from both rodents and humans suggests that GABA_A_R potentiation and EEG delta power are inversely related.

Finally, we suggest that our finding of stronger gamma oscillations in Dup15q syndrome as compared with nonsyndromic ASD may reflect a common mechanism for beta2 and gamma oscillations involving feedback inhibition between pyramidal cells and interneurons. This hypothesis is supported by the fact that beta2 power and gamma power were strongly correlated in all three cohorts. Nonetheless, the effect size observed in beta2 (*d* = 1.73) was greater than that of gamma (*d* = 0.97), suggesting that SBOs are a clearer biomarker of Dup15q syndrome than gamma oscillations.

### SBOs as markers of gene expression?

Although we did not measure *UBE3A* and GABA receptor gene expression from participants in our study, these data support the need for future investigations that can directly examine the relationship between EEG power and mRNA transcript levels from *GABRA5*, *GABRB3*, *GABRG3*, and *UBE3A*, all genes which are duplicated in Dup15q syndrome. In particular, there already exists evidence from pharmacological studies of GABA_A_R PAMs (i.e., benzodiazepines) [25–27], as well as correlations between motor evoked beta power and resting GABA levels [41], suggesting an important relationship between beta activity and GABAergic transmission. As with beta power in our study, Scoles et al. found greater mean and variance in neural *GABRB3* expression in a small cohort (*n* = 8) of postmortem tissue samples from individuals with Dup15q syndrome (isodicentric duplications) compared to nonsyndromic ASD and TD tissue samples [42]. The close resemblance between the distribution of *GABRB3* expression in the Scoles et al. study and the distribution of beta power in our study can be visualized in Fig 4B of Scoles et al. (2011) (*cf*. Fig 3, this paper). Slight overlap in distributions of beta power between the Dup15q syndrome cohort and the ASD comparison group could potentially reflect point mutations of GABA_A_R subunit genes in some children in the ASD comparison group. Finally, many studies of benzodiazepine GABA_A_R PAMs have shown reductions of delta power in a variety of contexts [27, 38–40, 43, 44], suggesting that reduced delta power in the wakeful spontaneous EEG of Dup15q syndrome participants could also be explained by GABA_A_R abnormalities such as altered subunit expression. Further work in humans and animal models will be necessary to test this hypothesis.

Another possible cause of reduced EEG delta power in Dup15q syndrome is duplications of *UBE3A*, the causative gene of Angelman syndrome. Patients with Angelman syndrome do not express *UBE3A* in neural tissue and show elevated EEG delta power [34, 35, 37], the opposite electrophysiological phenotype as Dup15q syndrome. For this reason, it is plausible that an inverse relationship exists between EEG delta power and *UBE3A* expression levels. It is also possible that *UBE3A* overexpression influences beta power, perhaps an indirect effect mediated through the GABAergic system. For instance, a recent study in a *UBE3A*-null mouse model found that loss of *UBE3A* can impair co-release of subcortical GABA, thus demonstrating their intrinsic functional relationship [37]. However, considering that SBOs have been reported in individuals with paternal Dup15q syndrome [6, 7], the paternally imprinted *UBE3A* alone cannot explain SBOs in Dup15q syndrome.

### SBOs and epilepsy risk

**Fig 6:**
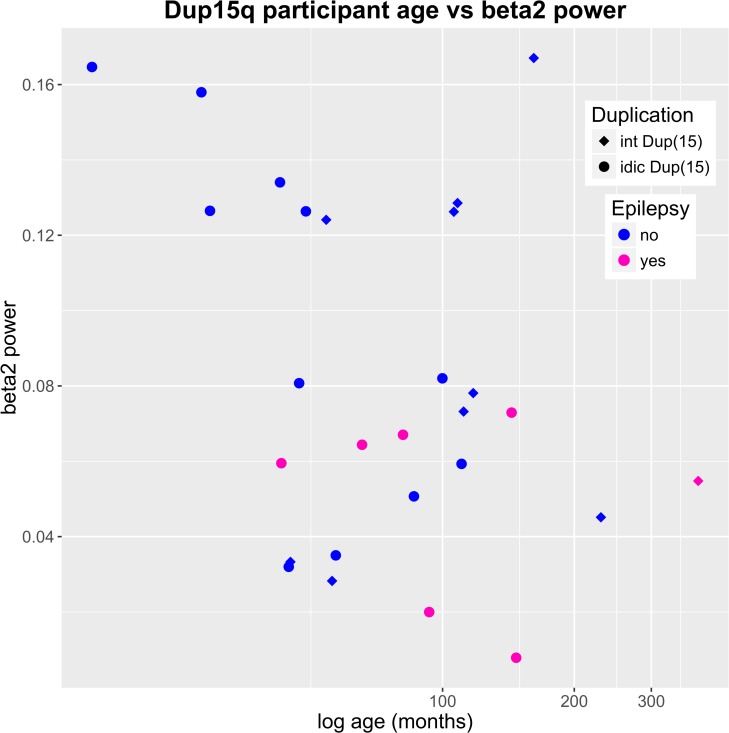
Dup15q syndrome participants by age, beta2 power, duplication type, and epilepsy status. A scatter plot of age versus beta2 power in Dup15q syndrome reveals a cluster of very young participants with very high beta2 power (top left corner). None of these participants have epilepsy. All participants with epilepsy feature beta2 power < 0.08. Note that the abscissa has been log-transformed to accommodate a large number of young participants and a much smaller number of older participants.

We identified an inverse relationship between SBOs, particularly beta2 power, and epilepsy in Dup15q syndrome in the larger cohort analysis. At first glance, this result could be interpreted as SBOs being markers of enhanced GABAergic tone (as found in GABAergic medications such as benzodiazepenes), thus providing neural protection against seizures in this population. However, interpretation of this relationship requires caution, as likely there are modifying factors such as background EEG, antiepileptics, and developmental level of individuals with epilepsy that may influence this relationship [45]. In particular, with our small sample, we could not fully disentangle the relationship between age and epilepsy. To better understand the relationship between age, duplication type, epilepsy status, and beta2 power, we visualized all 4 variables in Fig 6. Log-transformed age (abscissa) is plotted against beta2 power (ordinate), with interstitial duplications represented by diamonds and isodicentric duplications represented by circles. Children are color coded by epilepsy status (pink for epilepsy, blue for no epilepsy). As seen in this figure, those participants with the highest beta2 power included our youngest children without epilepsy. Of note, while it is difficult to separate the role of epilepsy *per se* from the impact of medications on EEG, anticonvulsant medications typically increase beta activity rather than decrease such activity [46]. Although no subjects in this study were treated with benzodiazepine or barbiturate medication, most children with Dup15q syndrome with epilepsy were treated with levetiracetam, which has been shown to increase (rather than decrease) relative beta power in epilepsy patients [46].

We must emphasize that the relationship between GABAergic activity and epilepsy is complex as is, most likely, the relationship between beta oscillations and epilepsy. It may seem paradoxical that a syndrome associated with overexpression of GABA receptor genes could also confer such a high risk for epilepsy. In general, synaptic, i.e. phasic, inhibition is mediated by α1 or α2 containing GABA receptors that also have a γ2 subunit producing an increase in chloride conductance favoring hyperpolarization. The extrasynaptic GABA receptors, usually α4 or α6 combined with δ (no gamma) are sensitive to small changes in ambient GABA, and they produce sustained lowering of the membrane potential [47]. Excessive GABAergic activity can produce two effects that are both potentially epileptogenic. First, the spike-wave bursts in the thalamo-cortical network are initiated by hyperpolarization, since the lowered membrane potential is the trigger for the low-threshold calcium currents [48]. Secondly, prolongation of inhibition can promote synchronization of such networks, which can occur with enhancement of tonic inhibition [49]. Thus spike-wave stupor can be treated with intravenous benzodiazepines which act only at synaptic receptors. On the other hand, generalized spike-wave paroxysms become more frequent and longer in duration when ambient GABA is increased by other medications.

### Limitations and future directions

The relatively small sample size, albeit representative of the full clinical spectrum of this rare disorder, does limit further subgroup analyses and examination of clinical correlates of this biomarker. The sample size also precludes the development of a multi-variable prediction model of these elevated beta oscillations. The association with epilepsy, in particular, warrants further investigation through two approaches. First, through longitudinal studies we can examine changes in EEG power after the onset of epilepsy and, by doing so, elucidate whether beta oscillations represent a protective biomarker for the development of seizures.

However, these findings have laid the foundation for a larger scale study of the functional and clinical implications of electrophysiological biomarkers in this syndrome. Through a multi-site, coordinated effort with the National Dup15q Alliance, we will expand our sample size to ask the following questions: First, do changes in state modulate beta power, in particular during cognitive or perceptual tasks, or during sleep? One might hypothesize that persistent beta power in sleep could disrupt sleep architecture enough to impact cognition and behavior in these children with neurodevelopmental disabilities. Moreover, a lack of modulation of EEG oscillations during cognitive tasks could directly hinder learning. We will directly examine the relationship between beta power and changes in beta power with more quantitative measures of cognition and autism severity. Second, what are the exact genetic underpinnings of this biomarker? We will examine electrophysiological markers in several pre-clinical models of Dup15q syndrome (full genetic duplication compared to *UBE3A* overexpression mouse). Translational studies linking gene expression and SBOs in individuals with Dup15q syndrome will elucidate the specific role of *UBE3A*, *GABRA5*, *GABRB3*, and *GABRG3* gene expression on this biomarker and help further understand the genetic effects on transcript levels that lead to the pathogenesis of Dup15q syndrome.

## Conclusions

The field of ASD research has sorely lacked quantifiable biomarkers that may help parse the neurobiological heterogeneity of this spectrum of disorders. Moreover, as genetic testing in ASD and related neurodevelopmental disorders becomes clinical gold standard, an increasing number of children are diagnosed with genetic variants that not only will elucidate causal mechanisms but also therapeutic targets [50]. The identification of these targets necessitates quantifiable biomarkers that relate directly to genetic mechanisms. Studies in Dup15q syndrome provide a promising path towards this mission, as the elucidation and quantification of an electrophysiological biomarker could improve diagnosis and prognostication, as well as measurement of target engagement and outcomes in clinical trials, a model that can inform similar investigations in the quickly expanding number of high-risk genetic syndromes associated with neurodevelopmental disorders.

## Abbreviations

Dup15q syndrome: Duplication 15q11.2-q13.1 syndrome
ASD: Autism spectrum disorder
EEG: Electroencephalogram
GABA_A_R: Gamma aminobutyric acid type A receptor
TD: Typically developing
ROIs: Regions of interest
SBOs: Spontaneous beta oscillations
CNVs: Copy number variants
MSEL: Mullen Scales of Early Learning
SB5: Stanford Binet Intelligence Scales-Fifth Edition
DAS II: The Differential Ability Scales Second Edition [: 15: ]
PLS-5: Preschool Language Scales-Fifth Edition
Leiter-R: Leiter International Performance Scales–Revised
FIR: Finite impulse response
EMG: Electromyogram
PCA: Principal component analysis
ICA: Independent component analysis
ICs: Independent components
PSDs: Power spectral densities
ANOVA: Analysis of variance
E/I balance: Balance between neural excitation and inhibition
PAMs: Positive allosteric modulators
